# Bootstrapping Q Methodology to Improve the Understanding of Human Perspectives

**DOI:** 10.1371/journal.pone.0148087

**Published:** 2016-02-04

**Authors:** Aiora Zabala, Unai Pascual

**Affiliations:** Department of Land Economy, University of Cambridge, Cambridge, United Kingdom; University of Illinois at Chicago College of Medicine, UNITED STATES

## Abstract

Q is a semi-qualitative methodology to identify typologies of perspectives. It is appropriate to address questions concerning diverse viewpoints, plurality of discourses, or participation processes across disciplines. Perspectives are interpreted based on rankings of a set of statements. These rankings are analysed using multivariate data reduction techniques in order to find similarities between respondents. Discussing the analytical process and looking for progress in Q methodology is becoming increasingly relevant. While its use is growing in social, health and environmental studies, the analytical process has received little attention in the last decades and it has not benefited from recent statistical and computational advances. Specifically, the standard procedure provides overall and arguably simplistic variability measures for perspectives and none of these measures are associated to individual statements, on which the interpretation is based. This paper presents an innovative approach of bootstrapping Q to obtain additional and more detailed measures of variability, which helps researchers understand better their data and the perspectives therein. This approach provides measures of variability that are specific to each statement and perspective, and additional measures that indicate the degree of certainty with which each respondent relates to each perspective. This supplementary information may add or subtract strength to particular arguments used to describe the perspectives. We illustrate and show the usefulness of this approach with an empirical example. The paper provides full details for other researchers to implement the bootstrap in Q studies with any data collection design.

## Introduction

Q is a powerful methodology (also known as Q technique or Q-sort) to shed light on complex issues in which human subjectivity is involved. Subjectivity is understood as how people conceive and communicate their point of view [1]. Q helps to identify different patterns of thought on a topic of interest, using a systematic procedure and an analytical process that is clearly structured and well established [2,3]. The method is considered semi-qualitative and is appropriate to investigate diversity of discourses or to facilitate public participation, for example.

Typically, in order to implement Q methodology, respondents express their views by sorting a set of statements from *most agree* to *most disagree*. This data collection method makes explicit the relative opinion of a respondent about every statement with respect to all other statements, therefore the interpreted perspectives are wholistic and integrate trade-offs. The data analysis reduces all responses to a few different factors, each factor being one perspective that represents those who share similar views. Results can be used in further research, for example, to prove hypotheses that correlate perspectives with behaviour or with other observed variables.

The use of Q method is growing remarkably across social, health, and environmental studies. It is used to identify typologies such as conservationist attitudes towards markets [4,5], farmer environmental perspectives [6], opinions about new environmental legislation [7], sustainability discourses [8], stakeholder views on energy from biomass [9], discourses on forest management [10], or citizen views on climate change policy [11]. However, the analytical process has received little attention in the last three decades, and further relevant information could be extracted from the data by making use of recent statistical and computational advances.

Few papers are concerned with exploring the analysis, internal validity, reliability, or external replicability of Q studies. Arguably, Fairweather [12,13] makes the most relevant contributions to the discussion of external and construct validity. He investigates the internal replicability of three studies by analysing sub-samples of responses and interpreting the sub-sample results in comparison to the results of the entire sample [13]. He finds that interpretation across sub-samples may change remarkably in solutions of more than two factors or in factors that have few highly representative respondents (*flagged* Q-sorts, see next section). Additionally, an extensive test-retest reliability study has demonstrated how some views may be more permanent than others [6].

Despite providing substantial evidence for discussion, these studies did not lead to improvements in the analytical process, and the bootstrap has not yet been applied to Q. Since Stephenson [14] described and discussed the *SE* of factor scores, and beyond the standard Q analysis detailed in Brown [2], no further procedures have been put forward to enhance the accuracy of the results to the authors' best knowledge. Current methodological debates focus on the methods for extraction and rotation of factors, but tend to regard all other analytical decisions as irrelevant or having little influence over the interpretation.

While this paper leaves aside the heated discussions about these two aspects, it focuses on the calculation of measures of variability and argues that this estimation should receive further attention because critical results depend upon them, and because of the limitations of the current standard estimates. In the analysis, the standard error (*SE*) is estimated as a unique value for all the statements within a factor, no confidence intervals (*CI*) can be calculated, and there is no measure of variability for the factor loadings—the values that correlate each respondent with the perspectives. This lack of uncertainty levels for all the results can be an area of concern, especially for researchers with quantitative background.

To address this gap in reporting the level of confidence of results, we propose a novel analytic approach of bootstrap re-sampling in Q. Bootstrapped—data-based—measures of variability are considered superior to assumption-based measures because bootstrap estimates do not assume normally distributed data [15]. We describe and illustrate how to estimate measures of variability specific to individual statements and to factor loadings. These measures of variability can help exploring the stability and reliability of perspectives within the conditions of the particular study and without the need to replicate it. The *SE* specific to each statement and the bootstrapped estimates of the results contribute with more precise information to the final interpretation of perspectives, which therefore could change slightly or thoroughly.

The key reasons to use the bootstrap in Q are threefold. It yields improved (more detailed and precise) estimates of values and *SE*s. It provides measures of variability for results that the standard analysis does not (either *SE* or *CI* for the values of respondents and of individual statements). These improved estimates and new measures of variability enhance the understanding of the data and of the level of confidence of the results, provide further support for key analytical decisions (such as flagging or deciding on the number of factors to extract), and may increase the accuracy of the interpretation. Finally, the bootstrap is less strict with violations of parametric assumptions, which may be encountered with Q data, such as non-continuity of responses or not normal distributions. Bootstrap results may be reported if these make a significant difference with respect to the standard results, if variability estimates are important for the interpretation, or to communicate results with higher detail and precision.

Next in this paper, we summarise the standard analytical process of Q and identify the key research decisions. Focusing on the first of these decisions, we explain the main theoretical considerations to implement the bootstrap in Q. We develop an algorithm for its implementation and provide general guidelines for its interpretation. This is followed by an application that shows how additional, relevant insights are obtained. With the details that the paper provides, other researchers can readily implement bootstrap in Q studies of any number of Q-sorts, of statements, and any distribution shapes. In addition, functions to run bootstrap in Q are available in the R package 'qmethod' [16]. The final section discusses benefits and limitations of this additional procedure, and suggests future directions for improvement.

## The Standard Approach in Q Method

**Fig 1:**
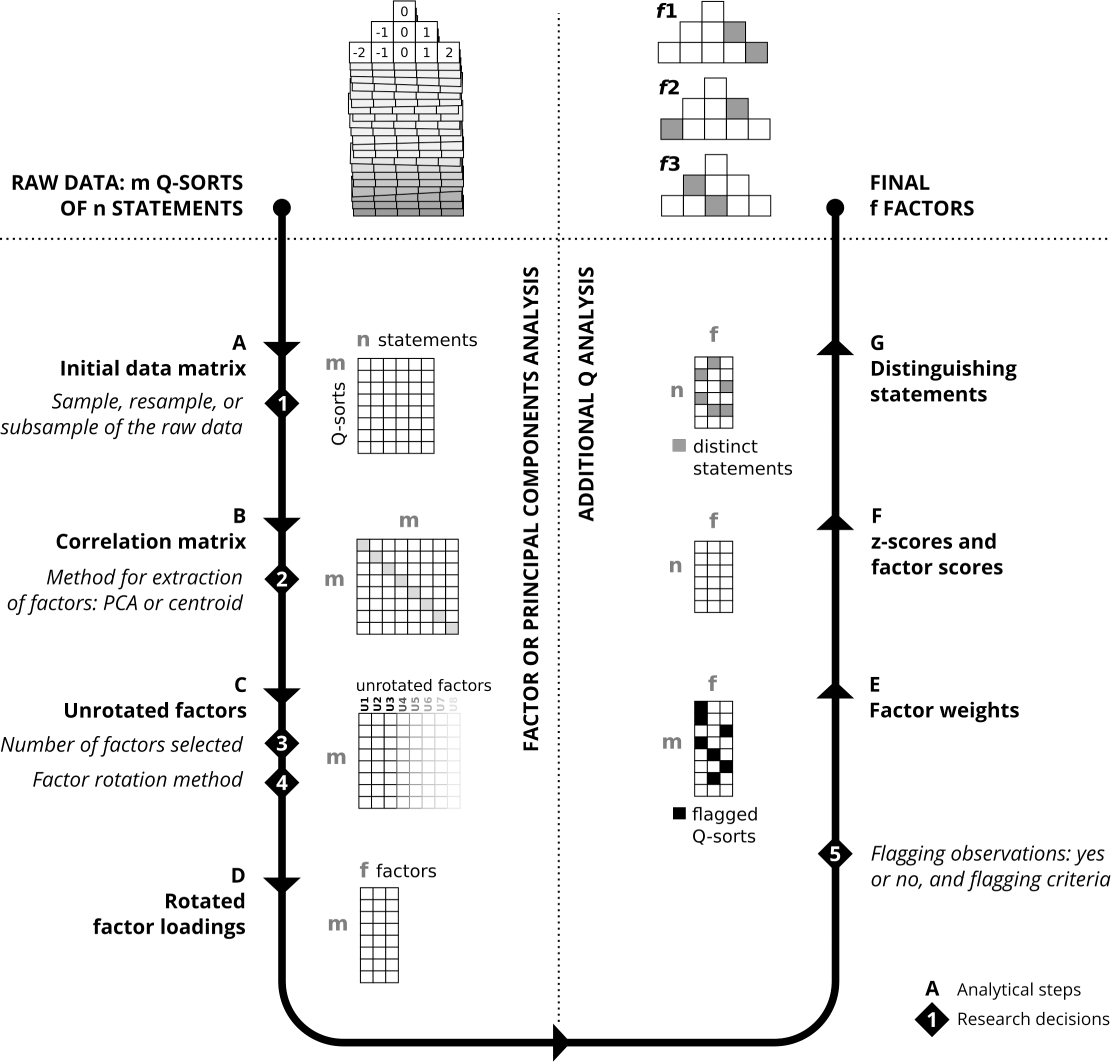
The standard analytical process in Q methodology.

In simplified terms, in order to conduct a Q study the researcher uses explicit criteria to select a set of statements from the *concourse*. The concourse is a hypothetical concept that conveys the infinite set of possible expressions that refer to a topic of concern, from all different points of view; it contains statements pertaining to multiple discourses [17]. The selected statements (typically between 40 and 80) are written on one card each, and these cards are given to respondents who rank them over a grid that represents a prearranged frequency distribution [18] (an example of such grid is shown on top of Fig 1). Respondents follow a *condition of instruction*, which frequently entails ranking the statements from *most agree* to *most disagree*. The grid is usually shaped as a quasi-normal distribution, based on the assumption that fewer statements are considered of highest agreement and of highest disagreement [2], although there is total flexibility with regards to its shape and size.

The methodology is exploratory because it is not focused on estimating the frequency and distribution of perspectives within a population, but rather on mapping the plurality of these perspectives, whether or not they are minority ones. The group of respondents is a representation of the population diversity rather than a representative sample of the population. This purposive sampling approach enables to uncover patterns that may not be detected otherwise because they may be unrelated to observable demographic characteristics [19]. Identifying all perspectives regardless of their proportional representation may be particularly important in research questions where highly influential individuals have a strong effect on others' opinion or behaviour, for example.

The analysis reduces the data to a few summarizing factors, based on principal components analysis (PCA) or centroid factor analysis (FA; centroid is a rare form of FA, used exclusively in Q methodology, which results are similar—but non-identical—to those of standard FA or PCA). Each factor is a perspective that represents respondents with similar views. When PCA is used, the term *component* would be more accurate, however in Q literature *factor* is generally used for either case, thus this paper follows the convention.

Abundant literature describes how to perform a complete Q study (e.g [18,20]) and most studies that use the method also explain it (e.g. [21]). Van Exel & de Graaf [22] and Watts & Stenner [23] are accessible options to start with, and Dziopa & Ahern [24] provide a structured outline of the key elements to be reported in a study, which may facilitate its assessment and replication. Brown [2] gives full details of the analytical process and Stenner et al. [25] and Brown et al. [19] provide a comprehensive historical account of the methodology, which dates back to 1935.

### The analytical process

The basic analytical principle is to correlate the entire responses of individuals. The process reduces the data to a few typical responses, based on PCA or centroid FA. Distinctively, instead of correlating variables (as in regular PCA and FA), in Q the respondents are correlated in order to elucidate relationships between them. The standard data-reduction method is followed by a set of analytical steps specific to Q methodology. The final results consist of a small number of sets of sorted statements (typically called the *factors*) that are different from each other and that synthesise the perspectives existing among respondents.

The key terms to understand the process of analysis in Q are *Q-sorts*, *factors*, *factor loadings*, *z-scores*, and *factor scores* (the precise naming can vary depending on the source). The distribution of statements by a single respondent—the response—is called a Q-sort. When ranking the statements according to the given condition of instruction, each statement is assigned a value that corresponds to the column in which the respondent places it. For example, in the distribution at the top left of Fig 1, the statement of most disagreement would receive a value of −2. A Q-sort is thus the array of values that a respondent implicitly gives to statements.

A factor is the weighted average Q-sort of a group of respondents that responded similarly, and it represents an archetypical perspective: how a hypothetical best-representative respondent of those with similar views would sort the statements. Although no respondent may be a perfect representative of a factor, typically each respondent is more similar to one factor than to the rest. The correlation of each Q-sort with each factor is given by the factor loadings, which range from −1 to +1. A respondent is most similar to the factor with which it has the highest loading.

The ranking of statements within each factor is given by the scores (z-scores and factor scores), which indicate a statement's relative position within the factor. The z-score is a weighted average of the values that the Q-sorts most closely related to the factor give to a statement, and it is continuous. Factor scores are integer values based on z-scores and they are used to reconstruct the Q-sort of a factor, which is then interpreted.

Fig 1 illustrates the analytical steps for a study of **m** number of Q-sorts and **n** number of statements as it is described in the literature [2]. It shows the steps necessary to analyse the raw Q-sorts (top left of Fig 1) in order to finally obtain the few summarizing factors built upon statement factor scores (top right of Fig 1). The process of analysis has two main parts: reducing data (steps A-D) and obtaining statement results (steps E-G).

#### From data to factors

The first part (steps A-D) is the standard data reduction in multivariate analysis. The data collected are structured in a two-dimensional matrix (step A) with statements and Q-sorts. Cell values in this matrix are the value of the column in the grid where the respondent placed the statement. Next, Q-sorts are correlated (instead of variables, as it is common in multivariate methods; B). From this correlation matrix, unrotated factors are extracted using PCA or centroid FA (C). Among the unrotated factors, the first few explain most of the variance of the initial correlation matrix and thus only a few factors are selected and rotated. Factors are rotated in order to make the data structure clearer. Rotation in Q can be mathematically optimal, such as varimax, or manual (judgemental), the latter occurring when the researcher has relevant knowledge about a given respondent. This step results in a matrix of factor loadings that correlate Q-sorts with the rotated factors (D).

The second part of the analysis (steps E-G) is specific to Q and reconstructs the archetypical response of each factor, based on the raw data and on the factor loadings. It consists of three steps: *flagging* the Q-sorts that will define each component (E), calculating the scores of statements for each factor (F), and finding the distinguishing and consensus statements (G).

Only the most representative Q-sorts for each factor are used for subsequent calculations; these Q-sorts are identified and *flagged* (E). The purpose of flagging is to maximise differences between factors [1] and it may be done either automatically or manually. Automatic pre-flagging is based on two criteria: that the loading is significantly high [2], and that the loading is much larger than the loadings of the same Q-sort for other factors; the square loading for a factor is higher than the sum of the square loadings for all other factors [26]. Some Q-sorts may be considered confounding because they load highly in more than one factor. Further flags can be manually added or eliminated after examining the loadings.

The z-scores (defined above) indicate the relationship between statements and factors: how much each factor *agrees* with a statement. Factor scores are obtained by ordering statements by z-score, and matching the statements to the array of possible values in the original distribution (F). For example in Fig 1, the array is (−2, −1, −1, 0, 0, 0, 1, 1, 2). In conventional distributions which reference values are zero in the central column, and negative and positive for disagreement and agreement respectively, the sign of the z- and factor scores approximately represents the *agreement* or *disagreement* of the given factor with the statement. Absolute magnitude of z- and factor scores indicate the salience of statements within a factor.

#### General characteristics of the factors

In addition, overall characteristics are obtained for each factor: the number of flagged Q-sorts, composite reliability (Eq 1), eigenvalues, percentage of explained variance, and *SE* of z-scores (Eq 2). The composite reliability of a factor *f* is calculated as [2]:

$$r_{f}=\frac{0.8\;p}{1+\left(p-1\right)0.8}$$ (1)

Where *p* is the number of Q-sorts flagged for the factor. The value 0.8 is the customary value used in Q methodology for the *average reliability coefficient*, which is the expected correlation between two responses given by the same person ([2], pp.211 and 244).

The *SE* of z-scores for a factor *f* is calculated as [2]:

$$SE_{f}=s_{f}\sqrt{1-r_{f}}$$ (2)

Where *s* is the standard deviation of the distribution (of the array of values of the grid).

Two additional matrices indicate the similarity between the z-scores of each pair of factors. These are the correlation coefficients and the standard errors of differences between factors *i* and *j* (*SED*_*ij*_, based on the *SE*) [2]:

$$SED_{ij}=\sqrt{SE_{i}^{2}+SE_{j}^{2}}$$ (3)

Both the composite reliability and the variability explained give indication of the strength of a factor, although they are rarely used for interpretation. However, the *SE* and *SED* are important because they determine between consensus and distinguishing statements (see below), and this is very frequently used in the interpretation. However, as seen in Eqs 1–3, all three indicators are based primarily on the number of defining Q-sorts (*p*) and no other indicator of variability—aside from the array of scores in the grid—is used to calculate them.

In the final step (G), the statements that distinguish factors and those that are consensus are identified, based on whether a statement's z-scores across factors are statistically different. A statement is distinguishing for a factor if it ranks in a position that significantly differs from its rank in other factors. The threshold for a difference to be considered significant is given by the *SED*_*ij*_ for each pair of factors (multiplied by 1.96 for p-value < .05, and 2.58 for p-value < .01) ([2], p.245). If the difference in z-scores is larger than the threshold, then the statement distinguishes factor *i* from *j*. The distinguishing statements and their position in the distribution are key to interpret the factor.

Those statements which are not distinguishing for any of the factors are *consensus*. Consensus statements may arise for various reasons, for example, they reveal what the common ground is among perspectives, they are ambiguous, or they are taboo and therefore respondents do not want to express engagement.

As seen above, the simplified calculation of the *SE* and *SED*, and the fact that Q studies are usually of small samples may raise concerns over the robustness or reliability of the numerical results, particularly among researchers with a quantitative background. The *SED* values are of high importance because they are used to determine distinguishing and consensus statements, therefore enhancements in their calculation may also improve the accuracy of results.

### Key methodological considerations

The numbered text in Fig 1 indicates the key decisions that a researcher makes along the standard analytical process. The sample used in the analysis (decision number 1) may (rarely) vary if some particular Q-sorts are excluded, or to implement internal replicability methods such as the ones explored by Fairweather [13] and the bootstrap presented in this paper. On the method for extraction of factors (2), there is discussion among practitioners of Q about whether to use PCA or the centroid method. Both are widely used and they yield similar results [1,18]. Regarding the decision on the number of factors to extract (3), the various possible criteria are extensively described in the Q and PCA literatures (more details are given in [18], and a synthetic summary in [21]). The following is a non-exhaustive list of criteria used to determine the number of factors: the variability explained by factors, at least two Q-sorts loading significantly, eigenvalues higher than a threshold, results from parallel analysis, visual inspection of the screeplot, the factor is theoretically relevant and meaningful, interpretability, and parsimony (which can be pre-assessed upon inspection of the correlation matrix between factors, highly correlated factors being very similar). The technique for rotation (4) depends on the aim and on the previous knowledge that the researcher has about respondents. It can be manual rotation if the researcher identifies one or a few important Q-sorts around which the rotation revolves. Otherwise, mathematically optimal solutions are used, such as varimax. These rotations may fit the data equally well, and a decision criteria can be to choose the rotation that results in higher interpretability. The final decision is whether to flag Q-sorts to calculate scores or instead whether to use all the Q-sorts (5). The former choice predominates because it yields more clearly distinctive factors, and flagging may be done automatically or manually.

## Methods: Bootstrapping Q

The bootstrap applies to the first decision in Fig 1, and it consists in drawing resamples from the original sample multiple times and in analysing each of these resamples [27]. A (non-parametric) resample is a random set of observations in which some observations from the original sample may be repeated and others may be absent. For example, given an initial sample of Q-sorts **m** = (*m*_*1*_, *m*_*2*_, *m*_*3*_, *m*_*4*_), a resample **m'** is drawn for each bootstrap repetition. The resample **m'** contains a random array of elements from **m** and, because the random selection is with replacement, a given element of **m** may not appear or may appear more than once in the resample, e.g. **m'** = (*m*_*1*_, *m*_*1*_, *m*_*1*_, *m*_*4*_). With each resample **m'**, a full analysis is performed. This process of resampling and analysing is repeated multiple times. The results from all the resamples for a given statistic (such as the factor loading for the first Q-sort and factor) constitute an estimation of the distribution of this statistic, from which relevant measures of centrality and of variability can be calculated (such as mean and standard deviation). These measures are alternative estimates of the results for the original sample.

The bootstrap is used across disciplines to obtain estimates for various results in PCA, such as eigenvalues and eigenvectors [28,29], loadings [30,31], and also to help deciding on the number of components to extract [32,33]. Some authors provide detailed explanations about bootstrapping PCA [15,34,35] and its performance is compared against other methods to estimate measures of variability, with a growing consensus about the benefits of using bootstrap in PCA [15,30]. The bootstrap is also considered an adequate approach to calculate standard errors for mathematically complicated processes—such as Q analysis—because it requires no theoretical calculations [27].

For Q, we suggest a non-parametric bootstrap resampling with replacement of Q-sorts, for three main reasons. First, we consider the bootstrap more adequate for Q than other methods of internal replicability, namely cross-validation and the jackknife [36], because it allows more repetitions with smaller samples—which is usually the case in Q studies—and therefore may provide more accurate estimation of *SE*s. The jackknife would be appropriate to assess highly influencing Q-sorts. Second, the assumptions of the non-parametric version of the bootstrap are less strict than those of the parametric version [15,35]. Third, we discard conducting the bootstrap by resampling statements for two main theoretical reasons specific to Q. A Q-sort is interpreted as a whole, and eliminating a statement would imply that the relative nature of the score given to each statement is lost. Besides, each step would miss statements from the whole set and, in the process of obtaining factor scores, matching the full array of scores to an incomplete array of statements would inflate the variability.

Q methodology datasets are generally small (usually below 80 respondents). While discussing the appropriateness of data-reduction techniques for small samples is beyond the scope of this paper (see, e.g. [37]), we explored the performance of bootstrapping Q under different sample sizes by means of simulation. Simulation results suggest that the bootstrap produces highly accurate estimates of the true measures of spread with samples of 45 respondents and above.

The higher the number of repetitions, the better the approximation will be to the true estimates. The number of repetitions may be limited by computing capability and the literature suggests that at least 50 repetitions are necessary and 200 are satisfactory to calculate *SE*s [27], and that at least 1,000 are necessary to estimate CIs [38]. A rule of thumb when bootstrapping PCA is to perform a number of repetitions at least 40 times the size of the sample [38].

With respect to other decisions in Q analysis, the extraction and rotation methods of choice are to be used consistently in all the bootstrap repetitions. We also suggest automatic flagging (or otherwise a fixed manual set of flags) for all the repetitions. This is because manually inspected flagging and manual rotation in each repetition may not be plausible due to computational limits and, arguably, to the incomparability of individually manipulated repetitions.

### The alignment problem

An essential consideration when bootstrapping PCA is that variability estimates can be arbitrarily inflated due to the *alignment problem* [31,35]. This problem occurs because factor extraction and rotation are purely mathematical procedures that overlook the underlying theoretical concept behind each factor. These mathematical procedures are aimed at fitting the sampled data optimally, therefore they may give different results when using slightly different data, as explained below.

The alignment problem is intuitively similar to the parameter identification problem in regression and it has two main consequences. First, axis reflection (or sign indeterminacy or sign swap) refers to the fact that factor loadings can arbitrarily change sign in subsequent bootstrap repetitions, even though the absolute magnitudes of the loadings remain within the underlying distribution space. A way of assessing whether reflection is a problem is by looking at histograms of bootstrapped factor loadings for a given observation and factor: they may be bimodal when the problem occurs [29]. Second, the factors extracted are usually ordered according to the percentage of variability that they explain. When two or more factors explain a similar amount of variability, axis reordering or interchange (order swap) may happen in some of the bootstrap repetitions. As a consequence, this issue would introduce values which do not belong to the underlying distribution.

In order to solve both sources of inflated variability, it is assumed that the underlying distribution is similar to that of the analysis of the initial sample and thus the results of each bootstrap repetition are inspected and corrected if necessary towards matching the initial results. A simple and robust solution is to reflect and reorder factors when necessary [31], which solve respectively the two sources of variability.

**Fig 2:**
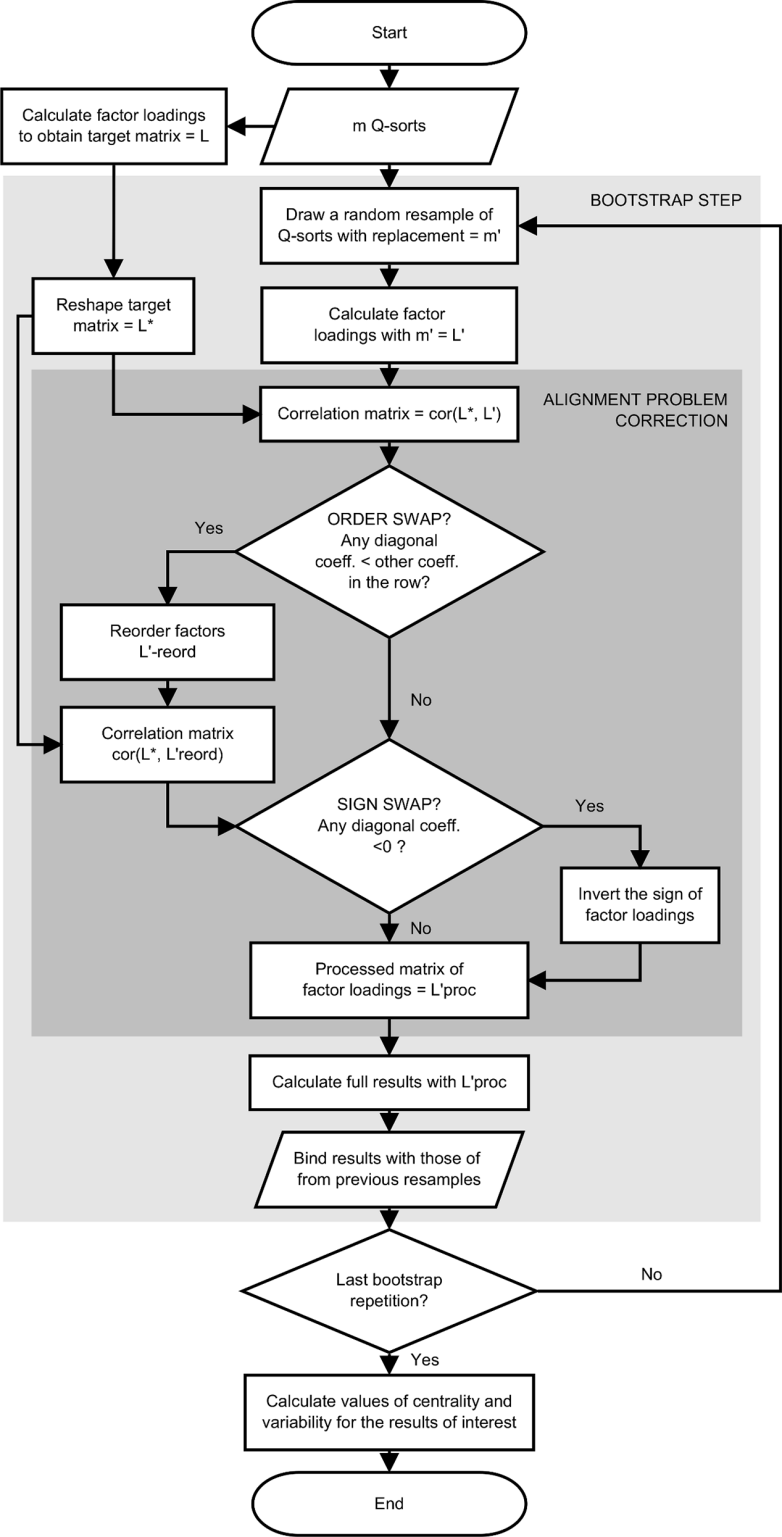
Algorithm for bootstrapping Q methodology.

We develop an algorithm to bootstrap Q as illustrated in Fig 2. This algorithm starts by performing the analysis with the original sample to obtain the target matrix of loadings, which is used subsequently to apply the corrections. For each bootstrap step, a resample is drawn, the factor loadings of the resample are calculated, and then the correction of the alignment problem is implemented (darkest area in Fig 2). This correction begins by building a correlation matrix between the loadings of the target matrix and the loadings of the bootstrapped resample. If no alignment problem occurred, the coefficients in the diagonal should all be positive and closer to 1 than all other coefficients.

To test for axis reordering for a given factor, the absolute correlation coefficients outside the diagonal are compared to the absolute coefficient in the diagonal. If the diagonal coefficient is smaller than any of the others, then factors need reordering. For the given position, the bootstrapped factor with the highest correlation coefficient is chosen. After reordering (if needed), a new correlation matrix is built between the reordered factors and the target factors. To test for axis reflection (sign swap), only the correlations in the diagonal are examined: negative coefficients indicate reflection and these are corrected by inverting the sign of the loadings in the bootstrapped factor.

Another alternative suggested to correct the alignment problem in PCA bootstrap is orthogonal Procrustes rotation for optimal reflection of the factor loadings, using the loadings of the initial sample as target matrix [35,39,40]. Reordering-reflection and Procrustes may also be used together [15]. In the experiments run for this paper, both approaches give similar results and Procrustes provide only slightly smaller variability measures. We suggest the reordering-reflection approach because it is more intuitive and transparent.

### Resampling the Q-sorts

As a consequence of resampling Q-sorts instead of statements, the bootstrap in Q presents its distinctiveness within the literature on the bootstrap in PCA. In order to implement bootstrap in PCA, typically observations are resampled and variables are correlated. The initial array of observations **o** = (*o*_*1*_, *o*_*2*_, *o*_*3*_, *o*_*4*_) is resampled into, e.g., **o'** = (*o*_*1*_, *o*_*1*_, *o*_*1*_, *o*_*4*_), while the variables **v** = (*v*_*1*_, *v*_*2*_, *v*_*3*_, *v*_*4*_) remain the same. The PCA of a resample **o'** begins by correlating variables and the extraction of components results in a matrix of component loadings with variables **v** as rows and factors as columns. In a bootstrap step, all the variables in **v** are represented and represented only once. The alignment problem is then corrected, using the loadings from the analysis of the initial sample as the target matrix. Each row in the target matrix corresponds to the same row in the resampled matrix of loadings.

By contrast, in bootstrapping Q analysis, respondents (Q-sorts) are correlated instead of variables. The initial array of Q-sorts **m** = (*m*_*1*_, *m*_*2*_, *m*_*3*_, *m*_*4*_) is resampled into, e.g. **m'** = (*m*_*1*_, *m*_*1*_, *m*_*1*_, *m*_*4*_). The extraction of factors results in a matrix of loadings with the resampled set **m'** of Q-sorts as rows and factors as columns. The corrections for the alignment problem compare the matrix of loadings from the resample **m'** and the target matrix **m** row by row, and they are sensitive to the order of the rows in each matrix. As a consequence of **m** ≠ **m'**, the resampled matrix of factor loadings is incomparable with the initial target matrix—by pairing different Q-sorts in the comparison, the correction methods would give spurious results. The solution that we adopt for bootstrapping Q is to reorder the rows in the target matrix in every bootstrap repetition, resulting in a target matrix with an adapted array of rows, e.g. m* = (*m*_*1*_, *m*_*1*_, *m*_*1*_, *m*_*4*_), so that m* = **m'**.

### Interpretation of the bootstrap results

The main statistics of interest estimated with the bootstrap are the z-score and its *SE* value for each statement and factor. The former is calculated as the mean of the z-scores obtained in all bootstrap iterations for a given statement and factor, and the latter is calculated as the standard deviation [27]. Additionally, in order to give an overall view of the internal robustness of the results, the bootstrap results may be compared with those of the initial sample in two ways. First, for a given statistic, the *bootstrap estimate of bias* is the difference between the value of the standard results and the bootstrap estimate. Second, whole factors may be compared by correlating the arrays of standard and bootstrap z-scores.

The *SE* of z-scores and, to a lesser extent, their bootstrap estimate of bias are useful to understand whether the position of a statement is stable. Also, this statement-specific *SE* allows researchers to conduct further inferential tests for determining more accurately than with the standard procedure whether a statement is distinguishing or consensus.

The bootstrap estimates of levels of confidence have two important potential consequences for the interpretation of results, depending on the position of unstable statements in the distribution and on whether they were considered as distinguishing in the standard analysis. First, the description of factors may be nuanced after increasing or decreasing the emphasis of certain statements. Second, in more severe cases the factors may be thoroughly altered due to key statements changing their position remarkably or showing large instability.

The position of a statement in a factor may be unstable or uncertain if either the z-score *SE* or the bootstrap estimate of bias of factor scores are large. In this situation, the opinion that those representing the factor have about this statement is ambiguous, thus its position in the factor may be unclear and this should be reflected in the interpretation. Likewise, statements that present very stable positions have a very reliable meaning; respondents' engagement with the statement is homogeneous within the factor in which statement is stable. If the interpretation is based only on factor scores, a stable statement can also be affected if the statements above or below in the factor ranking are unstable and with similar z-score values. Particular attention should be paid if any statements that are distinguishing in the standard results are unstable after the bootstrap. In order to synthesise this information for the interpretation, we suggest a classification of the most relevant statements according to their salience and stability (Table 1).

**Table 1:** Classification of statements in Q according to interpretative power.

		Stability (variability, *SE* of z-score)	
		High	Low
Salience (magnitude of z-score)	High	Highest interpretative power, very reliable	Meaningful within the factor but its relative position is fuzzy
	Low	Reliable but not particularly meaningful to interpret the factor	Lowest interpretative power, less reliable (although its instability and disengagement might have a relevant conceptual explanation)

Analogous to the interpretation of bootstrap estimates for statements, for Q-sorts the magnitude of the mean and the *SE* of factor loadings indicate respectively how much a respondent defines a factor and how stable it is as a definer. The frequency with which a Q-sort is flagged in the bootstrap is another measure of stability. A Q-sort may be an ambiguous representative of a factor if it is flagged in a medium proportion of the steps.

In sum, the following are possible sources of instabilities (and vice-versa for stabilities) to be detected with the bootstrap:

1. Unstable statements which *SE* is large or that change position in the factor.
2. Distinguishing statements that are not distinctive any more.
3. Ambiguous Q-sorts that are flagged inconsistently for a given factor. For example, if they are automatically flagged approximately between 20% and 80% of the bootstrap repetitions.

## Results and Discussion

The bootstrap can be implemented with any Q dataset and here we exemplify it with the well-known *Lipset* dataset with which Brown illustrates his detailed description of the analytical process in Q (Lipset 1963; Stephenson 1970, both in [2], p.205). In this dataset, *M* = 9 respondents placed *N* = 33 statements in a symmetric distribution with values ranging [−4, 4]. Stephenson drew these statements based on Lipset's study on value patterns on democracy. In his illustration, Brown extracts three factors using centroid extraction, manual rotation, and manual flagging.

For the bootstrap illustration, we perform Q analysis using PCA and varimax rotation to extract three factors, and we compare the results from a standard (full sample) analysis using PCA and varimax, with those from the bootstrap. The full Q analysis has been coded in R statistical language [16,41]. To validate the coding, the results of the standard analysis as implemented in R were contrasted with those obtained with the same options in PQMethod [42], a software commonly used for Q analysis. Both yield the exact same results at the three decimals (see details in [16]).

We use PCA for extraction because its computation is readily available in R and its results do not differ much from centroid extraction results (differences between both methods for the Lipset dataset for the factor loadings are of |.08| on average). We use varimax rotation because it is commonly used in Q studies and because different manual rotations in each repetition may raise concerns of incomparability. We draw 2,000 resamples and perform the full analysis for each of them, using the algorithm in Fig 2. Then we calculate the corresponding estimates and *SE* for all the statistics of interest.

### Q-sort loadings

The bootstrap results for Q-sorts (Table 2) show that the factor loading variability is outstandingly high for Q-sorts *FR9* and *US8* (for both, *SE* > .2 in all factors). The frequency of flagging is the fraction of the bootstrap repetitions in which the given Q-sort was automatically flagged (following the standard criteria, explained above). The high variability of these two Q-sorts is consistent with their ambiguous frequency of flagging in the bootstrap.

**Table 2:** Comparison of standard and bootstrap results for Q-sort factor loadings.

	Standard factor loading ^c^			Bootstrapped factor loadings (& *SE*) ^d^						Flagging frequency ^e^		
Q−sorts	F1	F2	F3	F1		F2		F3		F1	F2	F3
US1	.19	**.77**	−.17	.15	*(*.*14)*	.77	*(*.*20)*	−.20	*(*.*17)*	.01	**.91**	.06
US2	−.07	**.83**	.11	−.08	*(*.*12)*	.83	*(*.*16)*	−.02	*(*.*11)*	.00	**.98**	.02
US3	**.81**	−.02	−.09	.81	*(*.*15)*	−.01	*(*.*14)*	−.05	*(*.*15)*	**.98**	.01	.01
US4	**.78**	.23	.27	.76	*(*.*19)*	.19	*(*.*14)*	.20	*(*.*19)*	**.93**	.01	.03
JP5	**−.83**	.15	.03	−.84	*(*.*15)*	.14	*(*.*10)*	−.01	*(*.*11)*	**.96**	.01	.01
CA6	.15	−.18	**.88**	.11	*(*.*13)*	−.12	*(*.*20)*	.81	***(*.*21)***	.02	.08	**.87**
UK7	.14	−.35	**.74**	.11	*(*.*10)*	−.25	*(*.*19)*	.69	***(*.*31)***	.01	.13	**.83**
US8	−.09	**.66**	−.17	−.06	***(*.*21)***	.62	***(*.*25)***	−.22	***(*.*35)***	.04	.72	.22
FR9	.20	−.18	**−.45**	.16	***(*.*23)***	−.07	***(*.*22)***	−.56	***(*.*36)***	.11	.11	.59

Note: F, factors. Boldfaces:

^c^ flagged Q-sorts

^d^ *SE* >.2

^e^ frequency of flagging in the bootstrap >.8.

High flagging frequencies of seven Q-sorts indicate that these are clearly definers of the factors in which they are flagged. In contrast, *FR9* and *US8* are ambiguous representatives of one or more factors, because their frequency of flagging is spread across factors. High *SE* are also highlighted in the table, which coincide with the information extracted from the frequency of flagging. This information can help researchers to make analytical decisions when manual rotation or manual flagging are being considered. For example, Q-sorts with ambiguous flagging frequency may be excluded from flagging, or Q-sorts with small loading but also small *SE* could be flagged.

### Statement scores

**Fig 3:**
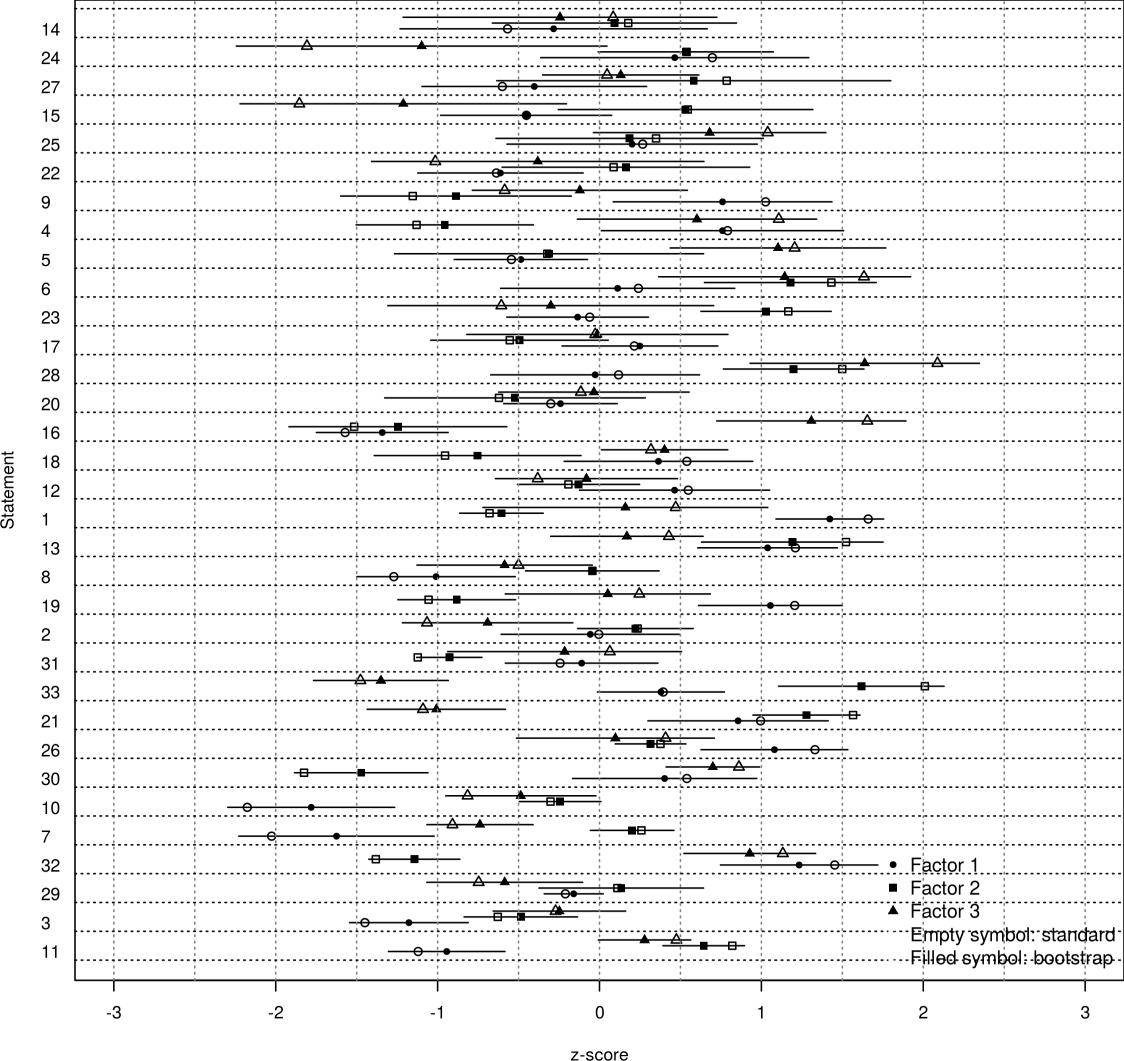
Statement z-scores: standard analysis, bootstrap estimates and *SE*.

Fig 3 shows the z-scores of the standard analysis, and the bootstrap estimates and the *SE* for statements. The bootstrapped z-scores are the means of z-scores of all 2,000 iterations and the *SE*s are the standard deviations. Following Table 1, the statements that are more powerful for interpretation are those with the highest absolute scores and smallest *SE* (e.g. statement n_1_ in factor 1). The statements are ranked in Fig 3 based on their cumulative *SE* for all the three factors. Statements at the bottom have smaller cumulative *SE* and so they are generally more stable.

The size of the error bar (which represents the *SE*) and its overlap with other error bars indicates whether the position of a statement is defined with precision. The comparison of the error bars of a statement within a given factor indicates whether the statement position in the factor is stable. Pairs of statements that have largely overlapping error bars may be interpreted as both being of similar salience. Similarly, comparing the error bars of the same statement across factors indicates whether the statement is distinguishing or not. Where the distribution of a statement is remarkably far from the distribution of the same statement in another factor, that statement distinguishes both factors. For example, even though the value of statement *n*_*24*_ for factor three is rather different from its value for the other two factors, the error bars are relatively large therefore its distinctiveness needs to be taken with due caution. By contrast, statement *n*_*25*_ is clearly a consensus statement. Statement *n*_*32*_ clearly distinguishes factor two from the other factors. In addition, the plot can help detecting whether most unstable statements do cluster in any side of the distribution, which could have a theoretical explanation. Also, the variability (*SE*) of a statement can be evenly distributed among the factors (e.g. statement *n*_*25*_), or the statement can be particularly unstable for a given factor (e.g. statement *n*_*24*_ for factor three), which could have a theoretical explanation.

Table 3 shows the bias estimates and the comparison of factor scores between the standard and the bootstrapped results. The estimate of bias of z-score (standard z-score minus bootstrapped z-score) is quite high for some statements (*z* > |0.20|), however this does not directly imply that these statements change position in the factor (factor scores). A statement's position also depends on the z-scores of statements which rank right above and below it, and on the size of the column with the same values in the grid. For example in the first factor, statement *n*_*7*_ with an estimate bias of 0.39 does not change its factor score, whereas statement *n*_*15*_ with an estimate bias of 0.00 changes one position due to the large bias estimate in a statement contiguous in the ranking, *n*_*14*_, which has a large *SE*. Arguably, there is not necessarily a strong correlation between the bias estimate and the size of the *SE*. Both values are complementary information for the interpretation of a statement.

**Table 3:** Comparison of bootstrap and standard results for statements.

Statement	z−score bias estimate^a^			Factor scores^b^											
	F1	F2	F3	F1 Sd.		F1 Bt.		F2 Sd.		F2 Bt.		F3 Sd.		F3 Bt.	
1	0.22	−0.07	**0.33**	4	**		**	−2	**			1	**		
2	0.03	0.01	**−0.37**	0				1				−3	**	**−2**	
3	**−0.26**	−0.15	−0.02	−3	*			−1				−1			
4	0.06	−0.16	**0.52**	2				−3	**		**	2			
5	−0.06	−0.03	0.08	−1		**−2**		−1				3	**		**
6	0.13	**0.27**	**0.49**	0	**		**	3				3			
7	**−0.39**	0.04	−0.17	−4	**		**	1	**		**	−2	**	**−3**	**
8	**−0.26**	−0.02	0.08	−3	*			0				−1		**−2**	
9	**0.23**	**−0.25**	**−0.48**	2	**		**	−3		**−2**		−1		**0**	
10	**−0.39**	−0.07	**−0.33**	−4	**		**	−1				−2			
11	−0.18	0.18	0.20	−2	**		**	2				2		**1**	
12	0.10	−0.06	**−0.32**	1				0				−1		**0**	
13	0.19	**0.33**	**0.26**	3				3				1	*		*
14	**−0.23**	0.10	**0.35**	−2		**−1**		0				0		**−1**	
15	0.00	0.00	**−0.65**	−1	*	**−2**		2	*		*	−4	**		
16	**−0.23**	**−0.26**	**0.37**	−3				−4				4	**		**
17	−0.01	−0.06	0.02	0				−1				0			
18	0.15	−0.19	−0.07	1				−2	**		**	1		**2**	
19	0.14	−0.18	0.20	3	*		*	−2	**		**	1	*	**0**	*
20	−0.05	−0.11	−0.05	−1				−1				0			
21	0.14	**0.28**	−0.08	2				4				−3	**		**
22	−0.04	−0.06	**−0.65**	−2				0				−2		**−1**	
23	0.07	0.14	**−0.33**	0		**−1**		2	**		**	−1			
24	**0.21**	0.00	**−0.73**	2				1				−4	**	**−3**	**
25	0.03	0.16	**0.36**	1				1				2			
26	**0.25**	0.06	**0.31**	3	*			1				1			
27	−0.16	0.20	−0.11	−2		**−1**		2				0		**1**	
28	0.13	**0.32**	**0.45**	0	**		**	3				4			
29	−0.05	−0.02	−0.18	−1				0				−2			
30	0.11	**−0.35**	0.16	1				−4	**		**	2			
31	−0.13	−0.20	**0.29**	−1		**0**		−2	*	**−3**		0		**−1**	
32	**0.23**	−0.22	**0.22**	4				−3	**		**	3			
33	0.02	**0.38**	−0.12	1	**		**	4	**		**	−3	**	**−4**	**

Note: Sd. *standard* factor scores, Bt. *bootstrap* factor scores (shown only if different from the standard result). Boldfaces:

^a^ bias estimates > |0.20| and

^b^ statements which position changes in the bootstrap. Significance of the distinctiveness of a statement:

* p < .01

** p < .05.

The comparison between standard and bootstrapped values shows that six, two, and fourteen statements have a different factor score respectively in each factor. From those statements selected as distinguishing in the standard analysis (with stars), one, one and five statements change their position respectively in each factor. Changes are all of just one position, which entails that in some cases the statement remains in the mid ground (e.g. statement *n*_*19*_ in factor three), that its salience is less emphasised (e.g. *n*_*2*_ in factor three), or that its salience is more emphasised (e.g. *n*_*31*_ in factor two). Also, applying the standard criteria to identify distinguishing statements over the bootstrap results reveals that, in general, less statements are distinguishing. The bootstrap confirms that some statements are very relevant for the interpretation (e.g. *n*_*1*_; or others that remain distinctive after the bootstrap), but other statements change their position in the distribution in such a way that they become less distinguishing (e.g. *n*_*7*_ between factors one and three).

In sum, statements that have small *SE* or that do not change their factor scores neither their classification as distinguishing or consensus are most reliable and can be used confidently in the interpretation. Statements that do not fulfil some of these conditions may be interpreted with due care, and this lack of reliability could also have a theoretical explanation. For example, if a statement has a large *SE* for a given factor, this indicates that those respondents within the given perspective do not have a homogeneous view about that statement (e.g. statement *n*_*24*_ in factor three). This additional information provides new valuable insights to interpret the perspectives.

## Conclusions

With the aim of elaborating more robust and reliable Q studies, this paper contributes to Q methodology by providing means to enhance the accuracy of the results. The paper explains how to calculate specific levels of confidence which the standard analysis does not offer, and provides guidelines on how to use this new information to improve the interpretation. To do so, we indicate where in the analytical process of Q researchers make decisions, in which sensitivity analyses can be performed. Focusing on the first of these decisions, the paper describes a novel implementation of the bootstrap in Q and explains important considerations specific to this particular case of the bootstrap in multivariate analysis. Details are given for the bootstrap to be implemented in Q studies of any number of Q-sorts, of statements, and any distribution shapes. The explanation is illustrated with an empirical application.

The bootstrap approach provides deeper and more accurate understanding of the data and of the robustness of perspectives, which may increase the confidence of researchers in the results. The approach quantifies the level of confidence associated to each statement and Q-sort for each of the factors. This information may nuance and in some cases change meaningfully the interpretation of perspectives with respect to an interpretation based on the standard results. Acknowledging ambiguity is particularly relevant if any of the statements selected as distinguishing in the standard analysis shows large variability after the bootstrap. On the contrary, statements that might initially be overlooked can present a very precise and distinguishing position in a given factor, hence become reliable definers of it.

Bootstrapping Q opens new methodological and empirical avenues for future research. This paper illustrates bootstrap with PCA and varimax rotation, yet the centroid method for the extraction of factors and manual flagging could potentially be implemented. The former involves further solvable computational complexity. The feasibility of the latter can be explored by applying a set of manually flagged Q-sorts that is fixed throughout the bootstrap. Also, expanding the bootstrap to a systematic sensitivity analysis by varying the number of factors (e.g. [26]) can help deciding on the number of factors to extract. This sensitivity analysis can shed light about the existing range of perspectives, by showing whether the factors excluded are sub-views of factors actually included or whether they are remarkably different and conceptually relevant. Additionally, an index that synthesises the stability information for each factor may be useful to compare factors within and across studies. Further, the performance of the bootstrap under different sample sizes may under different sampling circumstances may be investigated with further simulation studies. Last but not least, extensive empirical application of the bootstrap to several real datasets may help establishing acceptability thresholds.

Generally speaking, the process of analysis and interpretation of Q methodology can be enhanced using further quantitative developments. In addition to the bootstrap implemented here, other techniques in statistics have been put forward in the last decades (e.g. new methods to select the number of factors) and computational capacity has increased enormously, yet their application in Q is underexplored. These advances have a large potential to make Q a more solid and reliable method for the identification of the existing viewpoints and decision-making styles, in order to better understand and manage critical issues involving diverse human perspectives.
